# 1,3-Dibenzyl-1*H*-benzimidazol-2(3*H*)-one

**DOI:** 10.1107/S1600536811046071

**Published:** 2011-11-09

**Authors:** Youssef Kandri Rodi, Fouad Ouazzani Chahdi, El Mokhtar Essassi, Santiago V. Luis, Michael Bolte, Lahcen El Ammari

**Affiliations:** a Laboratoire de Chimie Organique Appliquée, Université Sidi Mohamed Ben Abdallah, Faculté des Sciences et Techniques, Route d’immouzzer, BP 2202 Fès, Morocco; b Laboratoire de Chimie Organique Hétérocyclique URAC21, Faculté des Sciences, Université Mohammed V-Agdal, Av. Ibn Battouta, BP 1014, Rabat, Morocco; c Departamento de Quimica Inorganica & Organica, ESTCE, Universitat Jaume I, E-12080 Castellon, Spain; d Institut für Anorganische Chemie, J.W. Goethe-Universität Frankfurt, Max-von-Laue-Strasse 7, 60438 Frankfurt/Main, Germany; e Laboratoire de Chimie du Solide Appliquée, Faculté des Sciences, Université Mohammed V-Agdal, Avenue Ibn Battouta, BP 1014, Rabat, Morocco

## Abstract

In the mol­ecular structure of the title compound, C_21_H_18_N_2_O, the fused-ring system is essentially planar, the largest deviation from the mean plane being 0.0121 (9) Å. The O atom and adjacent C atom are located in Wyckoff position 4*e* on a twofold axis (0, *y*, 1/4). The two benzyl groups are almost perpendicular to the benzimidazole plane, but point in opposite directions. The dihedral angle between the benzimidazole mean plane and the phenyl ring is 81.95 (5)°, whereas that between the two benzyl groups is 60.96 (7)°.

## Related literature

For pharmacological and biochemical properties of benzimidazoles, see: Gravatt *et al.* (1994 ▶); Horton *et al.* (2003 ▶); Kim *et al.* (1996 ▶); Roth *et al.* (1997 ▶). Ouzidan *et al.* (2011*a* ▶,*b* ▶,*c* ▶).
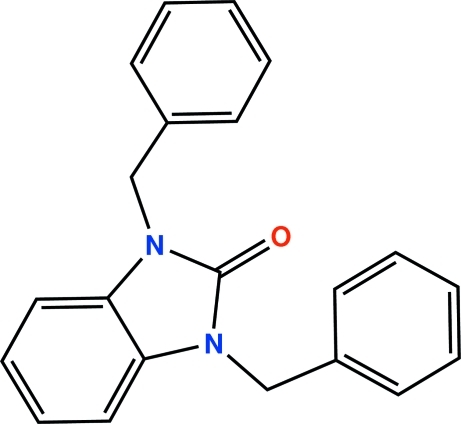


## Experimental

### 

#### Crystal data

C_21_H_18_N_2_O
*M* *_r_* = 314.37
Monoclinic, 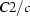

*a* = 19.5983 (7) Å
*b* = 9.0882 (2) Å
*c* = 10.0473 (3) Å
β = 115.593 (4)°
*V* = 1613.98 (10) Å^3^
*Z* = 4
Cu *K*α radiation
μ = 0.63 mm^−1^
*T* = 200 K
0.37 × 0.21 × 0.15 mm

#### Data collection

Agilent SuperNova Dual Cu at zero Atlas diffractometer
Absorption correction: multi-scan [*CrysAlis PRO* (Agilent, 2011) ▶, using spherical harmonics, implemented in SCALE3 ABSPACK scaling algorithm (Clark & Reid (1995 ▶)] *T*_min_ = 0.950, *T*_max_ = 1.000
7837 measured reflections
1611 independent reflections
1397 reflections with *I* > 2σ(*I*)
*R*_int_ = 0.028

#### Refinement

*R*[*F* ^2^ > 2σ(*F* ^2^)] = 0.037
*wR*(*F* ^2^) = 0.106
*S* = 1.07
1611 reflections
111 parameters
H-atom parameters constrained
Δρ_max_ = 0.15 e Å^−3^
Δρ_min_ = −0.16 e Å^−3^

### 

Data collection: *CrysAlis PRO* (Agilent, 2011 ▶); cell refinement: *CrysAlis PRO*; data reduction: *CrysAlis PRO*; program(s) used to solve structure: *SHELXS97* (Sheldrick, 2008 ▶); program(s) used to refine structure: *SHELXL97* (Sheldrick, 2008 ▶); molecular graphics: *XP* (Sheldrick, 2008 ▶); software used to prepare material for publication: *SHELXL97*.

## Supplementary Material

Crystal structure: contains datablock(s) I, global. DOI: 10.1107/S1600536811046071/im2334sup1.cif

Structure factors: contains datablock(s) I. DOI: 10.1107/S1600536811046071/im2334Isup2.hkl

Supplementary material file. DOI: 10.1107/S1600536811046071/im2334Isup3.cml

Additional supplementary materials:  crystallographic information; 3D view; checkCIF report

## Figures and Tables

**Fig. 1.**
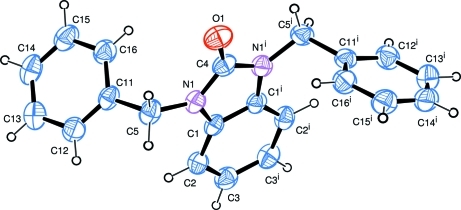
Molecular structure of the title compound with the atom-labelling scheme. Displacement ellipsoids are drawn at the 50% probability level. H atoms are represented as small circles.

## supplementary crystallographic information

### Comment

Benzimidazoles are very useful intermediates/subunits for the development of molecules of pharmaceutical or biological interest. Benzimidazole and its derivatives are an important class of bioactive molecules in the field of drugs and pharmaceuticals. Benzimidazole derivatives have found applications in diverse therapeutic areas including anti-ulcers, anti-hypertensives, anti-virals, anti-fungals, anti-cancers, (Gravatt *et al.* 1994; Horton *et al.* 2003; Kim *et al.* 1996; Roth *et al.* 1997).

As a continuation of our research work devoted to the development of substituted benzimidazol-2-one derivatives (Ouzidan *et al.*, 2011*a*, 2011*b*), we reported the synthesis of new benzimidazol-2-one derivative by action of benzyl chloride with 1*H*-benzimidazol-2(3*H*)-one in the presence of a catalytic quantity of tetra-n-butylammonium bromide under mild conditions to furnish two compounds: mono-substituted (Ouzidan *et al.*, 2011*c*) and the title compound (Scheme 1).

The title compound C_21_H_18_N_2_O is a new heterocyclic system deriving from benzimidazole. The crystal structure of this molecule is built up from two fused six and five-membered rings linked to two benzyl groups. The oxygen and the adjacent carbon atom are located in the Wyckoff position 4 e on the twofold axis (0, *y*, 1/4). The fused-ring system is essentially planar, with the maximum deviation of 0.0121 (9) Å for N1 as shown in Fig.1. The benzyl groups are almost perpendicular to the benzimidazole plane but oriented in opposite directions, with a dihedral angle of 81.95 (7). The dihedral angle between the two benzyl rings is abut 60.965 (7)°.

### Experimental

To a mxture of 1*H*-benzimidazol-2(3*H*)-one (0.2 g, 1.5 mmol), potassium carbonate (0.41 g, 3 mmol) and tetra-n-butylammonium bromide (0.05 g, 0.15 mmol) in DMF (15 ml) was added benzyl chloride (0.34 ml, 3 mmol). Stirring was continued at room temperature for 6 h. The salt was removed by filtration and the filtrate concentrated under reduced pressure. The residue was separated by chromatography on a column of silica gel with ethyl acetate/hexane (1/2) as eluent. The compound was recrystallized from ethanol to give colourless crystals (yield: 75%).

### Refinement

H atoms were located in a difference map and treated as riding with C—H = 0.93 Å for all H atoms with *U*_iso_(H) = 1.2 *U*_eq_for aromatic and methylene.

### Crystal data

**Table:**

C_21_H_18_N_2_O	*F*(000) = 664
*M**_r_* = 314.37	*D*_x_ = 1.294 Mg m^−^^3^
Monoclinic, *C*2/*c*	Cu *K*α radiation, λ = 1.54184 Å
Hall symbol: -C 2yc	Cell parameters from 5000 reflections
*a* = 19.5983 (7) Å	θ = 5–50°
*b* = 9.0882 (2) Å	µ = 0.63 mm^−^^1^
*c* = 10.0473 (3) Å	*T* = 200 K
β = 115.593 (4)°	Block, colourless
*V* = 1613.98 (10) Å^3^	0.37 × 0.21 × 0.15 mm
*Z* = 4	

### Data collection

**Table:**

Agilent SuperNova Dual Cu at zero Atlas diffractometer	1611 independent reflections
Radiation source: SuperNova (Cu) X-ray Source	1397 reflections with *I* > 2σ(*I*)
mirror	*R*_int_ = 0.028
Detector resolution: 10.4051 pixels mm^-1^	θ_max_ = 73.4°, θ_min_ = 5.0°
ω scans	*h* = −23→18
Absorption correction: multi-scan [*CrysAlis PRO* (Agilent, 2011), using spherical harmonics, implemented in SCALE3 ABSPACK scaling algorithm (Clark & Reid (1995)]	*k* = −11→11
*T*_min_ = 0.950, *T*_max_ = 1.000	*l* = −12→12
7837 measured reflections	

### Refinement

**Table:**

Refinement on *F*^2^	Secondary atom site location: difference Fourier map
Least-squares matrix: full	Hydrogen site location: inferred from neighbouring sites
*R*[*F*^2^ > 2σ(*F*^2^)] = 0.037	H-atom parameters constrained
*wR*(*F*^2^) = 0.106	*w* = 1/[σ^2^(*F*_o_^2^) + (0.0565*P*)^2^ + 0.4305*P*] where *P* = (*F*_o_^2^ + 2*F*_c_^2^)/3
*S* = 1.07	(Δ/σ)_max_ = 0.001
1611 reflections	Δρ_max_ = 0.15 e Å^−^^3^
111 parameters	Δρ_min_ = −0.16 e Å^−^^3^
0 restraints	Extinction correction: *SHELXL97* (Sheldrick, 2008), Fc^*^=kFc[1+0.001xFc^2^λ^3^/sin(2θ)]^-1/4^
Primary atom site location: structure-invariant direct methods	Extinction coefficient: 0.0010 (2)

### Special details

**Table:**

Experimental. CrysAlisPro, Agilent Technologies, Version 1.171.35.11 (release 16-05-2011 CrysAlis171 .NET) Empirical absorption correction using spherical harmonics, implemented in SCALE3 ABSPACK scaling algorithm (Clark & Reid (1995)).
Geometry. All e.s.d.'s (except the e.s.d. in the dihedral angle between two l.s. planes) are estimated using the full covariance matrix. The cell e.s.d.'s are taken into account individually in the estimation of e.s.d.'s in distances, angles and torsion angles; correlations between e.s.d.'s in cell parameters are only used when they are defined by crystal symmetry. An approximate (isotropic) treatment of cell e.s.d.'s is used for estimating e.s.d.'s involving l.s. planes.
Refinement. Refinement of *F*^2^ against all reflections. The weighted *R*-factor *wR* and goodness of fit *S* are based on *F*^2^, conventional *R*-factors *R* are based on *F*, with *F* set to zero for negative *F*^2^. The threshold expression of *F*^2^ > σ(*F*^2^) is used only for calculating *R*-factors(gt) *etc*. and is not relevant to the choice of reflections for refinement. *R*-factors based on *F*^2^ are statistically about twice as large as those based on *F*, and *R*- factors based on all data will be even larger.

### Fractional atomic coordinates and isotropic or equivalent isotropic displacement parameters (Å^2^)

**Table:**

	*x*	*y*	*z*	*U*_iso_*/*U*_eq_	
O1	0.5000	0.58201 (13)	0.2500	0.0492 (4)	
N1	0.53746 (5)	0.35600 (11)	0.19370 (10)	0.0366 (3)	
C1	0.52407 (6)	0.20943 (12)	0.21516 (11)	0.0333 (3)	
C2	0.54925 (7)	0.08014 (14)	0.17958 (13)	0.0390 (3)	
H2	0.5835	0.0806	0.1346	0.047*	
C3	0.52422 (7)	−0.05071 (14)	0.21593 (14)	0.0432 (3)	
H3	0.5402	−0.1444	0.1907	0.052*	
C4	0.5000	0.44731 (19)	0.2500	0.0378 (4)	
C5	0.58741 (7)	0.40758 (14)	0.13068 (13)	0.0412 (3)	
H5A	0.5785	0.5117	0.1088	0.049*	
H5B	0.5751	0.3564	0.0384	0.049*	
C11	0.67024 (7)	0.38418 (13)	0.23142 (12)	0.0376 (3)	
C12	0.71696 (8)	0.30976 (15)	0.18389 (14)	0.0459 (3)	
H12	0.6970	0.2694	0.0896	0.055*	
C13	0.79366 (8)	0.29464 (16)	0.27566 (17)	0.0522 (4)	
H13	0.8249	0.2450	0.2425	0.063*	
C14	0.82333 (8)	0.35325 (17)	0.41560 (16)	0.0518 (4)	
H14	0.8748	0.3445	0.4765	0.062*	
C15	0.77703 (8)	0.42476 (16)	0.46569 (14)	0.0507 (4)	
H15	0.7970	0.4626	0.5610	0.061*	
C16	0.70099 (8)	0.44026 (15)	0.37424 (14)	0.0450 (3)	
H16	0.6699	0.4888	0.4085	0.054*	

### Atomic displacement parameters (Å^2^)

**Table:**

	*U*^11^	*U*^22^	*U*^33^	*U*^12^	*U*^13^	*U*^23^
O1	0.0546 (8)	0.0361 (7)	0.0477 (7)	0.000	0.0136 (6)	0.000
N1	0.0302 (5)	0.0396 (6)	0.0357 (5)	−0.0023 (4)	0.0103 (4)	0.0015 (4)
C1	0.0233 (5)	0.0391 (6)	0.0293 (5)	−0.0018 (4)	0.0036 (4)	0.0003 (4)
C2	0.0295 (6)	0.0461 (7)	0.0374 (6)	0.0012 (5)	0.0107 (5)	−0.0031 (5)
C3	0.0371 (7)	0.0390 (6)	0.0469 (7)	0.0015 (5)	0.0120 (6)	−0.0036 (5)
C4	0.0329 (9)	0.0390 (9)	0.0311 (8)	0.000	0.0038 (7)	0.000
C5	0.0356 (7)	0.0493 (7)	0.0337 (6)	−0.0049 (5)	0.0101 (5)	0.0064 (5)
C11	0.0331 (6)	0.0406 (6)	0.0349 (6)	−0.0063 (5)	0.0106 (5)	0.0064 (5)
C12	0.0424 (7)	0.0531 (8)	0.0415 (6)	−0.0052 (6)	0.0174 (6)	0.0003 (6)
C13	0.0403 (8)	0.0581 (9)	0.0592 (8)	0.0011 (6)	0.0224 (7)	0.0053 (7)
C14	0.0336 (7)	0.0607 (9)	0.0511 (8)	−0.0050 (6)	0.0088 (6)	0.0129 (6)
C15	0.0412 (7)	0.0618 (9)	0.0377 (7)	−0.0089 (6)	0.0065 (6)	0.0011 (6)
C16	0.0393 (7)	0.0527 (7)	0.0397 (7)	−0.0028 (6)	0.0139 (6)	0.0000 (6)

### Geometric parameters (Å, °)

**Table:**

O1—C4	1.224 (2)	C5—H5B	0.9700
N1—C4	1.3807 (14)	C11—C12	1.3790 (19)
N1—C1	1.3924 (15)	C11—C16	1.3910 (18)
N1—C5	1.4541 (15)	C12—C13	1.3896 (19)
C1—C2	1.3801 (17)	C12—H12	0.9300
C1—C1^i^	1.397 (2)	C13—C14	1.376 (2)
C2—C3	1.3939 (18)	C13—H13	0.9300
C2—H2	0.9576	C14—C15	1.376 (2)
C3—C3^i^	1.390 (3)	C14—H14	0.9300
C3—H3	0.9773	C15—C16	1.3790 (18)
C4—N1^i^	1.3807 (14)	C15—H15	0.9300
C5—C11	1.5118 (17)	C16—H16	0.9300
C5—H5A	0.9700		
			
C4—N1—C1	110.02 (10)	H5A—C5—H5B	107.8
C4—N1—C5	124.18 (11)	C12—C11—C16	118.71 (12)
C1—N1—C5	125.68 (10)	C12—C11—C5	121.24 (11)
C2—C1—N1	131.45 (11)	C16—C11—C5	120.04 (12)
C2—C1—C1^i^	121.63 (7)	C11—C12—C13	120.56 (12)
N1—C1—C1^i^	106.92 (6)	C11—C12—H12	119.7
C1—C2—C3	116.92 (12)	C13—C12—H12	119.7
C1—C2—H2	121.4	C14—C13—C12	119.89 (14)
C3—C2—H2	121.7	C14—C13—H13	120.1
C3^i^—C3—C2	121.44 (8)	C12—C13—H13	120.1
C3^i^—C3—H3	119.4	C13—C14—C15	120.15 (13)
C2—C3—H3	119.1	C13—C14—H14	119.9
O1—C4—N1^i^	126.94 (7)	C15—C14—H14	119.9
O1—C4—N1	126.94 (7)	C14—C15—C16	119.88 (13)
N1^i^—C4—N1	106.12 (14)	C14—C15—H15	120.1
N1—C5—C11	113.16 (9)	C16—C15—H15	120.1
N1—C5—H5A	108.9	C15—C16—C11	120.78 (13)
C11—C5—H5A	108.9	C15—C16—H16	119.6
N1—C5—H5B	108.9	C11—C16—H16	119.6
C11—C5—H5B	108.9		

Symmetry codes: (i) −*x*+1, *y*, −*z*+1/2.
